# Evaluation des facteurs de risque cardio-vasculaire chez les imams: exemple des imams de Dakar (à propos de 67 cas)

**DOI:** 10.11604/pamj.2022.43.81.31883

**Published:** 2022-10-14

**Authors:** Abakar Bachar, Soumaila Nikièma, Désiré Massimbo, Brahim Nassour, Mamadou Leye, Aida Thiam, Maboury Diao, Sara Boury Gning, Cherif Mboup, Mustapha Sarr

**Affiliations:** 1 Hôpital Ibn Sina, Service de Cardiologie B, Rabat, Maroc,; 2 Hôpital Militaire d´Instruction Mohammed V de Rabat, Service de Cardiologie, Rabat, Maroc,; 3 Université El Hadji Ibrahima Niasse, Dakar, Senegal

**Keywords:** Evaluation, facteurs de risque cardio-vasculaire, imams, Dakar, Evaluation, cardiovascular risk factors, imams, Dakar

## Abstract

La prévalence des facteurs de risque cardio-vasculaire dans la population générale est peu étudiée dans les pays en voie de développement. L´objectif de ce travail est d´évaluer la prévalence des facteurs de risque cardio-vasculaire chez un groupe de population du Sénégal à savoir les imams. Nous avons mené une étude observationnelle transversale et descriptive qui s´est déroulée du 18 avril au 2 mai 2015. Etait inclus tout sujet faisant office d´imam, membre de l´association des imams et oulémas de Dakar. La population étudiée était relativement des personnes âgées avec 67,05 ans ± 12,35 d´âge moyen. Le niveau socio-économique était relativement bas dans l´ensemble de la population. La prévalence des facteurs de risque était beaucoup plus élevée chez les imams non rémunérés. L´enquête a révélé une prévalence élevée des facteurs de risque cardio-vasculaire avec au premier rang les dyslipidémies, observées dans 80% (n: 48) des cas. La prévalence des autres facteurs de risque était: la sédentarité (74,6% soit n: 50), hypertension artérielle (HTA) (56,7% soit n: 38), le diabète (17% soit n: 10), obésité (10,4% soit n: 7) et syndrome métabolique (19,4% soit n: 13). Les associations de facteurs de risque étaient fréquentes et 74,6% (n: 50) des imams enquêtés cumulaient au moins trois facteurs de risque cardio-vasculaire. Le risque cardio-vasculaire global était faible à modéré dans 76,66% (n: 51) des cas, élevé et très élevé dans 23,33% (n: 16) des cas. Cette enquête a révélé, d´une part, une prévalence élevée des facteurs de risque cardiovasculaire chez les imams et, d´autre part, que ces facteurs de risque ne sont pas souvent connus du sujet. Il est impératif de promouvoir la recherche concernant la prévention des facteurs de risque cardio-vasculaire.

## Abstract (en)

The prevalence of cardiovascular risk factors in the general population has been poorly studied in developing countries. The purpose of this study was to evaluate the prevalence of cardiovascular risk factors in a population group in Senegal (the imams). We conducted a cross-sectional and descriptive observational study from April 18^th^ to May 2^nd^, 2015. Any subject acting as an imam, member of the association of imams and ulamas of Dakar, was included. The study population was quite old, with a mean age of 67.05 ± 12.35 years. The socio-economic level was quite low in the overall population. The prevalence of risk factors was much higher among unpaid imams. The survey revealed a high prevalence of cardiovascular risk factors, with dyslipidemias as a major contributing risk factor observed in 80% of cases. The prevalence of other risk factors was: sedentary lifestyle (74.6%), hypertension (56.7%), diabetes (17%), obesity (10.4%) and metabolic syndrome (19.4%). Risk factor combinations were common and 74.6% of the imams surveyed had at least three cardiovascular risk factors. Global cardiovascular risk was low to moderate in 76.66% of cases, high and very high in 23.33% of cases. This survey showed, on the one hand, a high prevalence of cardiovascular risk factors in imams and, on the other hand, that these risk factors are not often known to subjects. It is imperative to promote studies for the prevention of cardiovascular risk factors.

## Introduction

Les maladies non transmissibles (MNT) telles que les maladies cardiovasculaires sont la première cause de morbidité et de mortalité dans le monde. Selon l´OMS, en 2014 on estime à 17,5 millions le nombre de décès imputables aux maladies cardio-vasculaires, soit 31% de la mortalité mondiale totale [1]. Plus des trois quarts des décès liés aux maladies cardio-vasculaires interviennent dans des pays à revenu faible ou intermédiaire [1]. La fréquence des facteurs de risque cardiovasculaire, et la forte mortalité qui en découle, ont conduit les praticiens regroupés au sein de sociétés savantes à élaborer des stratégies diagnostiques, thérapeutiques et préventives [2]. Ces stratégies ont mené à la notion de risque cardio-vasculaire global, dont l´évaluation doit reposer sur des méthodes reconnues, fondées sur des études épidémiologiques solides [2].

La prévalence des facteurs de risque cardio-vasculaire dans la population générale est peu étudiée dans les pays en voie de développement. Au Sénégal, une étude préliminaire réalisée en milieu rural en 1998 (880 sujets examinés) [3] situait les maladies cardio-vasculaires au deuxième rang après la pathologie infectieuse. Une autre enquête réalisée sur la population générale de Saint-Louis en 2011 a révélé une prévalence élevée des facteurs de risque cardio-vasculaire avec au premier rang les dyslipidémies, observées dans 64,6% des cas [4]. Les imams sont des guides religieux musulmans, responsables de la direction de la prière à la mosquée et de l´enseignement coranique. Ils ont une forte influence sur la communauté au Sénégal. Ainsi, nous avons décidé de mener une enquête portant sur l´évaluation des facteurs de risque cardio-vasculaire au sein de cette population spécifique (imams). Le but de ce travail était de contribuer à la réduction de la morbidité et de la mortalité liées aux affections cardio-vasculaires chez les imams. Les objectifs de ce travail étaient donc d´évaluer la prévalence des facteurs de risque cardio-vasculaire, de déterminer les facteurs de risque cardio-vasculaire en fonction des caractéristiques sociodémographiques et enfin et d´évaluer le risque cardio-vasculaire global selon plusieurs méthodes.

## Méthodes

**Type et période d´étude:** il s´agit d´une étude d´observation, transversale et descriptive qui a été menée du 18 avril au 2 mai 2015.

**Population d´étude:** l´enquête a porté sur l´ensemble des imams adhérant à l´association des imams et oulémas résidant dans la ville de Dakar.

**Critères d´inclusion:** a été inclus, tout imam, membre de l´association des imams et oulémas de Dakar.

**Critères de non inclusion:** tout imam qui n´était pas présent le jour de l´enquête.

**Outil et techniques de collecte des données:** l´outil de collecte était une fiche d´enquête (Annex 1) conçue pour cette étude, en tenant compte du questionnaire STEPS de l´Organisation mondiale de la Santé, comportant les variables sociodémographiques, anthropométriques, médicales, biologiques. Un questionnaire a été administré à chaque imam répondant aux critères d´inclusion. La pression artérielle était mesurée au niveau des deux bras chez tous les imams au siège de l´association des imams, en position assise, après un repos de 10 minutes au minimum. Le tour de taille était mesuré à mi-distance entre le rebord costal inferieur et l´épine iliaque antéro-supérieure. La pesée était effectuée grâce à un pèse-personne placé sur une surface stable et plane chez une personne légèrement vêtue, non chaussée et le résultat exprimé en kilogrammes. La mesure de la taille, en centimètres, a été effectuée à l´aide d´un mètre ruban chez des imams non chaussés et ne portant pas de chapeau. L´électrocardiogramme a été réalisé en position couchée, avec un électrocardiographe de marque Schiller. Les prélèvements biologiques avaient été effectués après un jeûne effectif de 12 heures.

**Définition opérationnelle des variables:** les six facteurs de risque cardio-vasculaire considérés dans cette étude étaient: le diabète, l´HTA, les dyslipidémies, l´obésité, la sédentarité et l´existence d´antécédents familiaux cardio-vasculaires (HTA, cardiopathie, AVC). Sédentarité: la sédentarité était définie par l´absence d´activité physique quotidienne ou la présence d´une activité physique d´une durée < 150 minutes par semaine. Hypertension artérielle: l´HTA a été définie par une tension artérielle supérieure ou égale 140/90 mm Hg et /ou un terrain connu d´hypertension artérielle. La classification de l´Organisation Mondiale de la Santé de 1999 a été retenue pour la stratification de la sévérité de l´hypertension artérielle.

**Evaluation du risque cardio-vasculaire global:** pour l´évaluation du risque cardio-vasculaire global, nous avons utilisé trois méthodes: la méthode de sommation, la méthode de Framingham - la méthode de l´OMS/IHS pour l´Afrique de l´ouest.

**Traitement des données et analyses statistiques:** les données recueillies ont été saisies grâce au logiciel Epi info version 3.5.2. L´analyse de la base de données a fait appel au module *analysis* du logiciel Epi info. Les graphiques ont été confectionnés grâce au logiciel Excel version 2007. La description a permis de calculer les fréquences pour les variables qualitatives et les moyennes avec leur écart type pour les variables quantitatives.

**Aspects éthiques:** le consentement libre et éclairé de chaque personne a été obtenu avant l´administration du questionnaire.

## Résultats

L´effectif de notre population était de 67 imams.

**Données sociodémographiques:** l´âge moyen des imams était de 67,05 ± 12,35 ans avec des extrêmes de 32 et 108 ans. La tranche d´âge de 65 à 74 ans était la plus représentée avec 34% (n=23). Plus de la moitié des imams résidaient en ville soit 55% (n=37). La proportion des imams scolarisés était de 43,3% (n=29). Tous les imams savaient lire et écrire en arabe. Trois quart des imams étaient sans profession, soit 75%, suivis des ouvriers (10%) et commerçants (5%).

### Les facteurs de risque cardio-vasculaire

**Figure 1:**
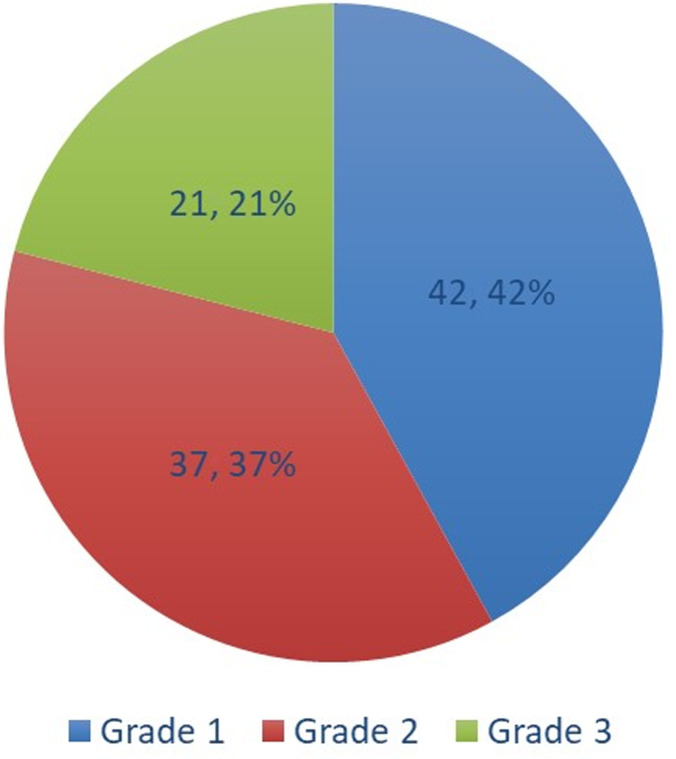
répartition des hypertendus selon le grade de l´HTA (OMS) (n=67)

**Figure 2:**
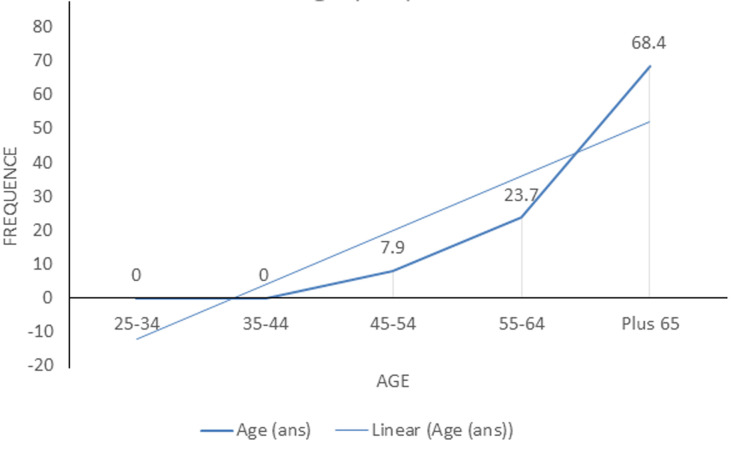
fréquence de l´HTA en fonction de l´âge

**L´hypertension artérielle:** la prévalence de l´HTA était de 56,7% (IC à 95% = 44, 0-68, 8), soit 38 imams. Chez les hypertendus, la pression artérielle systolique (PAS) moyenne était de 161,05 ± 14,8 mmHg (extrêmes: 140 et 200 mmHg). La pression artérielle diastolique (PAD) moyenne était 94,4 ± 9,7 mmHg (extrêmes: 70 et 110 mmHg) chez les hypertendus (Figure 1, Figure 2).

**Diabète:** la prévalence du diabète chez les imams était de 17% (IC à 95%=8,3-28,5) soit 10 imams. Chez les diabétiques connus (n=7), la glycémie moyenne était de 2,1 g/l avec un écart type de 0,74 g/l (Tableau 1).

**Tableau 1:** répartition de la glycémie en fonction du statut connu ou non de diabète (n=10)

Glycémie	Diabétiques connus n (%)	Diabétiques non connus n (%)	Total
<1.08 g/l	1 (14.3%)	45 (84.9%)	46
1.08-1.25 g/l	0 (0%)	5 (9.4%)	5
1.26-1.99 g/l	2 (28.6%)	3 (5.7%)	5
≥ 2 g/l	4 (57.1%)	0 (0%)	4

**Figure 3:**
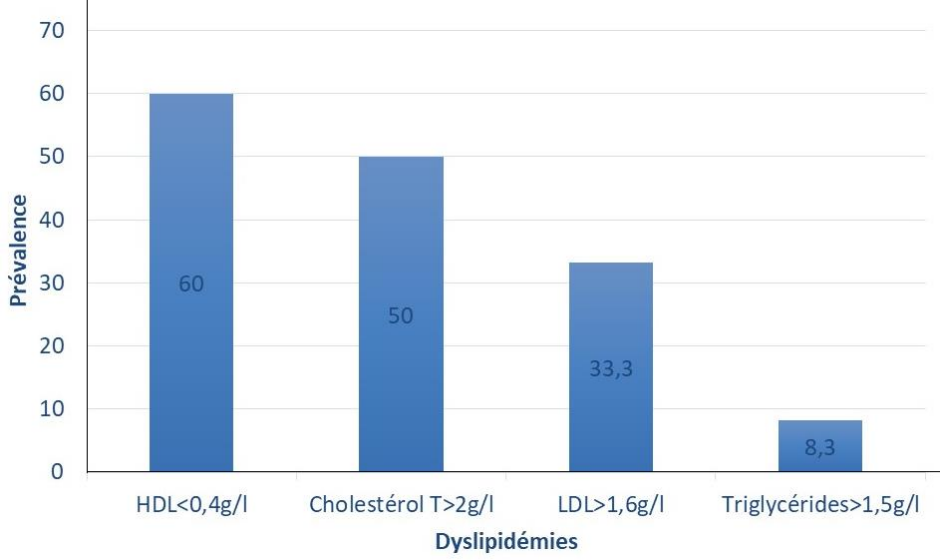
prévalence des différents types de dyslipidémie (n=60)

**La dyslipidémie:** la prévalence de la dyslipidémie chez les imams était de 80% (IC à 95%=67,7-89,2), soit 48 personnes. La dyslipidémie la plus fréquente était l´hypoHDLcholestérolémie avec une prévalence de 61% (n=36) suivie de l´hypercholestérolémie totale, 50% (n=30) et l´hyperLDLcholestérolémie 34% (n=20) (Figure 3).

**Figure 4:**
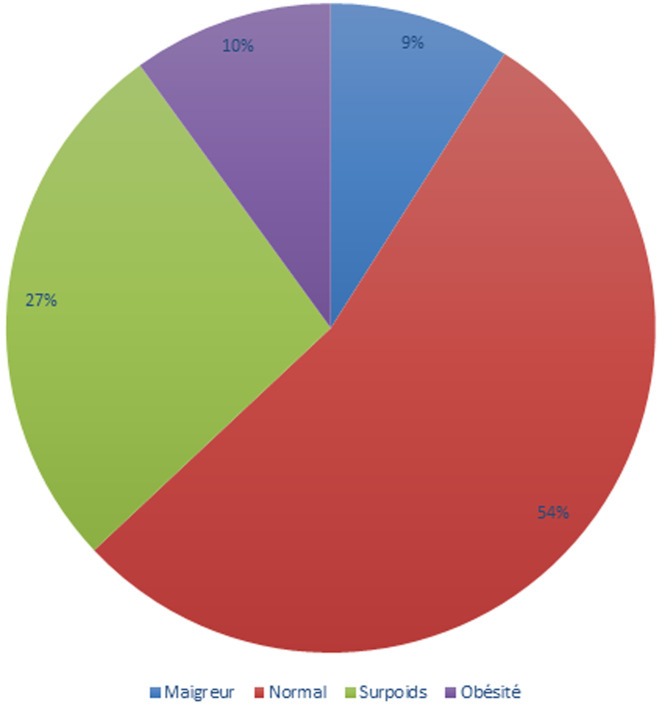
répartition des imams selon l´état pondéral

**L´obésité:** la prévalence de l´obésité était de 10,4%, soit 7 imams. L´IMC moyen était de 23,7 ± 4,3 kg/m^2^ (extrêmes: 15,6 et 39,2 kg/m^2^). Le périmètre abdominal moyen était de 89,5 ± 16,3 cm avec des (extrêmes: 66 et 183 cm). La prévalence de l´obésité abdominale était de 26,9% (IC à 95%=16,8-39,1) suivant la définition de l´IDF (Figure 4).

**La sédentarité:** la prévalence de la sédentarité chez les imams était de 74,6% (IC=62,5-84,5%), soit 50 imams.

**Le syndrome métabolique:** la prévalence du syndrome métabolique était de 19,4 %, soit 13 imams.

### Les complications

**Au niveau rénal:** la créatininémie moyenne de la population étudiée était de 10,57 ± 1,98 mg/l (extrêmes 7,1 et 16,4 mg/l). La moyenne de la clairance de la créatininémie était de 94,8 ± 20,6 avec des extrêmes de 52,6 et 145,9.

**Au niveau cardiaque:** l´électrocardiogramme (ECG) était enregistré chez tous les imams. Il était anormal chez 36 imams, soit 53,73% et normal chez 31 imams, soit 46,26%. Nous avions noté 10 cas d´hypertrophie ventriculaire gauche (HVG), soit 27,77% des imams et 12 cas d´hypertrophie auriculaire gauche, soit 33,33%. Les troubles de la conduction ont été retrouvés chez 14 imams (soit 38,88%), suivi des troubles du rythme (16,66%) et des troubles de la repolarisation.

### Evaluation du risque cardio-vasculaire global

**Selon la méthode de sommation:** le risque cardio-vasculaire était faible dans 25,4%, modéré dans 62,7% et élevé dans 11,9% de la population globale. La majorité des imams avait au moins 3 facteurs de risque cardio-vasculaire associés. Le nombre moyen de facteurs de risque était de 3,29 ± 1,6.

**Selon la méthode de Framingham:** le risque cardio-vasculaire à 10 ans selon la méthode de Framingham était faible dans 50%, moyen dans 31,66% et élevé dans 18,3%.

**Selon la méthode OMS/ISH:** le risque cardio-vasculaire à 10 ans selon la méthode OMS/ISH était faible dans 56,66%, moyen dans 20% et élevé à très élevé dans 23,33% (Tableau 2, Tableau 3).

**Tableau 2:** association des facteurs de risque cardio-vasculaire

Facteurs de risque cardiovasculaire (FDRCV)	Fréquence	Pourcentage	Intervalle de confiance
0 FDRCV	1	1.5 %	0% - 8%
1 FDRCV	3	4.5 %	0.9% - 12.5%
2 FDRCV	13	19.4 %	10.8% - 30.9%
3 FDRCV	17	25.4 %	15.5% - 37.5%
4 FDRCV	25	37.3 %	25.8% - 50%
5 FDRCV	7	10.4 %	4.3% - 20.3%
6 FDRCV	1	1.5 %	0% - 8%

**Tableau 3:** répartition du risque cardio-vasculaire par la méthode de sommation

Risque	Fréquence	Pourcentage	Intervalle de confiance
Faible	17	25.4%	15.5%-37.5%
Moyen	42	62.7%	50%-74.2%
Elevé	8	11.9%	5.3%-22.2%

## Discussion

Notre étude a permis d´évaluer les facteurs de risque cardio-vasculaire chez les imams de Dakar. La présente enquête a utilisé la méthode step-wise de l´OMS [5], mais avec quelques spécificités puisque nous avons réalisé l´ECG à tous les imams. Nous avons aussi utilisé une glycémie veineuse et avons dosé toutes les fractions lipidiques contrairement aux autres études réalisées en Afrique qui ont utilisé la glycémie capillaire et parfois dosé seulement le cholestérol total [6]. L´âge moyen de notre population d´étude était de 67,05 ans avec une proportion très forte des sujets âgés de plus de 60 ans, comparable à une étude récente réalisée à l´Hôpital principal de Dakar [7] où il était de 69,9 ans. L´âge élevé pourrait s´expliquer par le caractère spécifique de notre population d´étude.

Le diabète de type 2 est la forme la plus courante après 40 ans dans toutes les populations. Son évolution peut rester silencieuse pendant de longues années. C´est pourquoi le diagnostic est souvent tardif en dehors du dépistage systématique. La prévalence du diabète trouvée dans notre étude est de 17% comparable à une étude récente réalisée à l´Hôpital principal de Dakar [7] où elle était de 19,7%. Les études STEPS réalisées dans les autres pays africains retrouvaient une prévalence de 6 à 16% [8]. Les études réalisées à l´Hôpital Aristide le Dantec [2] placent les dyslipidémies au premier rang des facteurs de risque cardio-vasculaire. Ceci est conforme aux résultats retrouvés dans notre étude qui les place aussi au premier rang avec un taux de 80%. Contrairement au taux de prévalence de l´HTA chez l´adulte en milieu urbain et suburbain sénégalais estimé entre 18 et 27,3% dans le passé [3], la présente étude réalisée plusieurs années après observe un taux de prévalence de l´HTA de 56,7% similaire à une étude faite en Tanzanie 53,1% chez les hommes en milieu urbain [9]. Les différentes méthodologies de prise de la pression artérielle, et les définitions de l´HTA appliquées peuvent expliquer en partie la différence des taux de prévalence d´HTA.

En effet, les anciens travaux sur l´HTA utilisaient des tensiomètres anéroïdes, des sphygmomanomètres à mercure non standardisés et l´ancienne définition de l´OMS (165/95 mm Hg). Or, la présente étude a utilisé des sphygmomanomètres automatiques et la définition de l´OMS/ISH (140/90 mm Hg). Malgré tout, ce résultat laisse prédire l´augmentation de la prévalence de l´HTA au Sénégal. Les changements de modes de vie de la population sont les premiers facteurs à incriminer devant cette évolution épidémiologique. La prévalence de l´obésité et du surpoids dans notre étude était respectivement de 10% et 27%. La prévalence de l´obésité est proche de celle retrouvée à Brazzaville (18%) [6]. En Europe, 15 à 20% des adultes sont obèses et 35,9% sont en surpoids [10]. Notre étude a permis de noter que la sédentarité arrive au deuxième rang des facteurs de risque cardio-vasculaire avec un taux de 74,6%. Cette prévalence est légèrement supérieure à celle de Saint de Louis (64,7%) [4]. Cette prévalence élevée dans notre étude peut être expliquée par l´âge moyen (67,05 ans) et la pratique quotidienne de la majorité de nos imams (l´enseignement coranique, les prières). Il existe plusieurs définitions du syndrome métabolique et les prévalences varient en fonction de la définition utilisée. Ainsi, en utilisant la définition de l´IDF (*International Diabetes Federation*), nous avons retrouvé une prévalence de 19,4%.

Concernant les complications de l´HTA, nous avons noté qu´un peu plus du quart des imams hypertendus (27,77%) présentaient une hypertrophie ventriculaire gauche et 33,33% présentaient une hypertrophie auriculaire gauche. Ce taux est comparable à une étude récente réalisée à l´Hôpital principal de Dakar [7] où elle était de 28,85%. Les troubles du rythme étaient retrouvés dans 16,66% des cas. Ils étaient dominés par les extrasystoles ventriculaires. Les troubles de la conduction étaient retrouvés dans 38,88% des cas. Leur fréquence peut simplement être liée à l´âge. La fonction rénale était modérément altérée dans 3,3% des cas et une insuffisance rénale débutante était retrouvée dans 45% des cas.

Quant au risque cardiovasculaire global, il était faible dans 25,4%, modéré dans 62,7% et élevé dans 11,9% de la population globale. La majorité des imams avait au moins 3 facteurs de risque cardio-vasculaire associés. Ceci est probablement lié à un taux d´obésité et de surpoids qui sont élevés, des habitudes toxiques à savoir un mode d´alimentation déséquilibré, une activité physique insuffisante et des facteurs de stress psychosociaux chez les imams. Selon le modèle OMS/ISH qui a été conçu pour être appliqué en Afrique, la majorité des imams avait un risque faible à modéré dans 76,66% des cas et 23,33% avaient un risque élevé et très élevé. L´intérêt de notre travail repose sur la population cible de l´étude qui a une forte influence sur la communauté sénégalaise et pourrait contribuer à une sensibilisation sur le risque cardiovasculaire. Cependant, notre étude est limitée par la petite taille de l´effectif, la mesure de la pression artérielle qui s´est faite lors d´un seul passage contrairement aux recommandations européennes.

## Conclusion

Les maladies non transmissibles (MNT) telles que les maladies cardiovasculaires sont des causes majeures de morbidité et de mortalité dans le monde. A l´instar des pays développés où elles sont responsables d´une lourde morbimortalité, les maladies cardio-vasculaires sont émergentes en Afrique et au Sénégal en particulier. Au Sénégal, en 2014, les maladies cardio-vasculaires ont été classées première cause de mortalité selon le ministère de la santé. C´est dans ce contexte que nous avons décidé de mener une enquête portant sur l´évaluation des facteurs de risque cardio-vasculaire au sein d´une population spécifique (imams). Le but de ce travail était de contribuer à la réduction de la morbidité et de la mortalité liées aux affections cardio-vasculaires chez les imams. L´enquête a révélé d´une part la méconnaissance du risque cardiovasculaire par les populations, et d´autre part une prévalence élevée des facteurs de risque cardiovasculaire avec au premier rang les dyslipidémies, observées dans 80% des cas.

### Etat des connaissances sur le sujet

- Les maladies cardiovasculaires sont la première cause de morbidité et de mortalité dans le monde;
- Plus des trois quarts des décès liés aux maladies cardio-vasculaires interviennent dans des pays à revenu faible ou intermédiaire;
- Une autre enquête réalisée sur la population générale de Saint-Louis en 2011 a révélé une prévalence élevée des facteurs de risque cardio-vasculaire avec au premier rang les dyslipidémies.

### Contribution de notre étude à la connaissance

- La méconnaissance des facteurs de risque cardiovasculaire par les imams au Sénégal;
- Une prevalence élevée des facteurs de risque cardiovasculaire parmi les imams au Sénégal au premier desquels les dyslipidémies.

## Annex

**Annex 1**: fiche d´enquête (PDF - 407Kb)
